# Fecal source tracking and eDNA profiling in an urban creek following an extreme rain event

**DOI:** 10.1038/s41598-018-32680-z

**Published:** 2018-09-26

**Authors:** Zachery R. Staley, Jun Dennis Chuong, Stephen J. Hill, Josey Grabuski, Shadi Shokralla, Mehrdad Hajibabaei, Thomas A. Edge

**Affiliations:** 1 Environment and Climate Change Canada, Canada Center for Inland Waters, Burlington, ON L7S 1A1 Canada; 2 Memorial University, Department of Ocean Sciences, St. John’s, NL A1C 5S7 Canada; 3 University of Guelph, Centre for Biodiversity Genomics & Department of Integrative Biology, Guelph, ON N1G 2W1 Canada

## Abstract

Fecal contamination of recreational waters (i.e. lakes, rivers, beaches) poses an on-going problem for environmental and public health. Heavy rainfall can exacerbate existing problems with fecal contamination. As there could be variable sources of fecal contamination, identifying the source is critical for remediation efforts. This study utilized microbial source tracking (MST), chemical source tracking (CST) markers and environmental DNA (eDNA) metabarcoding to profile sampling areas and identify sources of fecal contamination in creek, stormwater outfall and beach sites in the Etobicoke Creek watershed (Toronto, ON). Water samples were collected before and immediately following an extreme rain event. MST and CST identified stormwater outfalls as an important source of human fecal contamination during dry and wet conditions. eDNA metabarcoding allowed for potential identification of additional sources of fecal contamination and provided additional evidence of human fecal contamination. The extreme rainfall event altered the eDNA profiles, causing creek and beach sites to reflect a greater diversity of mammal and bird eDNA sequences. The profiles provided by eDNA metabarcoding provide a proof of concept suggesting that eDNA metabarcoding can be a useful tool to complement MST and CST methods for profiling sources of fecal contamination and studying impacts of extreme rain events.

## Introduction

Poor water quality within recreational waters (i.e. any water body where swimming or water-related recreation occurs, such as lakes, rivers and beaches) is an on-going problem, especially in regions surrounding the Great Lakes designated as Areas of Concern (AOCs). In particular, many beaches within AOCs have had chronic problems with fecal contamination, resulting in beach postings and potential risks to public health^1^. Several studies have shown correlations between elevated levels of fecal contamination and increased risk of contracting gastrointestinal illness^2–4^. Often, rain events tend to exacerbate existing problems and elevate the impacts of nonpoint sources of contamination, such as increasing stormwater flow^5–7^.

Recreational water quality continues to be assessed using concentrations of fecal indicator bacteria (FIB), such as *Escherichia coli* and enterococci^8,9^. However, the sole use of FIB to assess water quality has limitations, such as providing no indication of the source(s) of fecal contamination, which can hinder remediation efforts and lead to erroneous conclusions regarding actual health risks^10^. To combat deficiencies linked to using only FIB concentrations to assess water quality, fecal source tracking (FST) methods have also been used to determine the source(s) of contamination in recreational waters^11^. Among the most commonly used FST methods is microbial source tracking (MST), which utilizes host-specific microbial DNA markers and has been used to identify multiple sources of contamination aside from human sewage (i.e., gull, dog, cow)^12–14^. MST methods therefore allow for the detection of specific, targeted sources. Additionally, chemical source tracking (CST) markers, such as caffeine, carbamazepine, codeine, cotinine and acetaminophen, have been identified as markers of human sewage contamination^15–20^.

Environmental DNA (eDNA) is the DNA extracted from environmental samples (e.g. water, soil) and it can provide biodiversity information through high throughput sequencing (HTS) analysis of marker genes such as species-specific DNA barcodes. This eDNA metabarcoding approach has predominantly been utilized in ecology and evolutionary studies of community dynamics. Additionally, it has been used to detect invasive species and as a method for characterizing communities of macroorganisms, including wildlife, birds, and amphibians^21–24^. Studies have shown that this method can have advantages, with an increased capacity to detect species which are either difficult to observe or present in low abundance^23,25^. Concentrations of eDNA have been shown, in many cases, to correlate with the relative abundance of their respective source^21,24^. eDNA in the environment degrades rapidly, within days to weeks, and tends to be relatively concurrent with the associated species^23,26^; however, the rate of eDNA decay is highly dependent on environmental conditions^27,28^. While most studies have used this method to examine the relative abundance of animal species in a given watershed, we explore eDNA analysis as a potentially useful tool to complement MST methods. In general, eDNA metabarcoding could provide a more comprehensive indication of all potential sources of fecal contamination within a watershed, whereas qPCR-based methods will only allow for detection of specifically targeted species.

**Figure 1:**
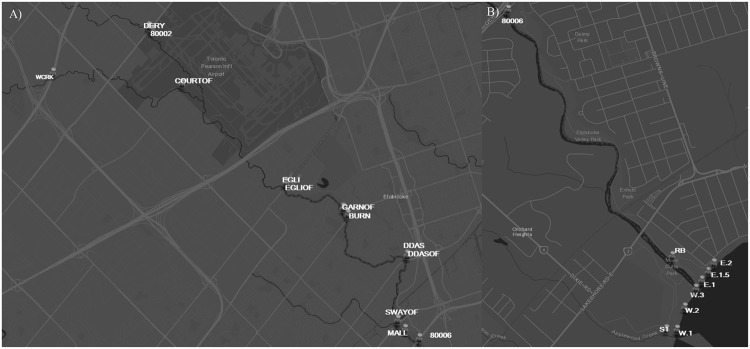
Map of sampling sites in (**A**) the upper Etobicoke Creek watershed and (**B**) at Marie Curtis beach. This map was created using ArcGIS updated in December 2017 (http://www.arcgis.com/home/index.html).

In this study, several FST methods were applied and *E*. *coli* were quantified at creek and stormwater outfall sites in the Etobicoke Creek watershed and at Marie Curtis Beach in the Toronto and Region AOC (Fig. 1). These FST methods were used to compare water quality associated with an extreme rain event in Toronto in 2013 with two sampling dates leading up to this extreme event. The extreme rain event occurred on July 8, 2013 when more than 126 mm of rain fell within 24 hours, most falling within about two hours. This was a new daily rainfall record for Toronto Pearson International Airport (dating back to 1937), as well as new records for 30-minute, and 1, 2, 6 and 12 hour rainfall totals at the airport, all in excess of 100-year return periods^29,30^. Microbial and chemical source tracking methods were applied, and eDNA metabarcoding analysis was used to profile human, mammal, frog, bird and fish species DNA sequences at each sampling site. The purpose of this study was to examine whether all FST methods were consistent in their identification of fecal contamination sources, particularly human sewage. Additionally, we aimed to assess whether eDNA metabarcoding could provide additional information regarding other potential fecal pollution sources which MST and CST methods could not.

## Results

### E. coli Enumeration

**Figure 2:**
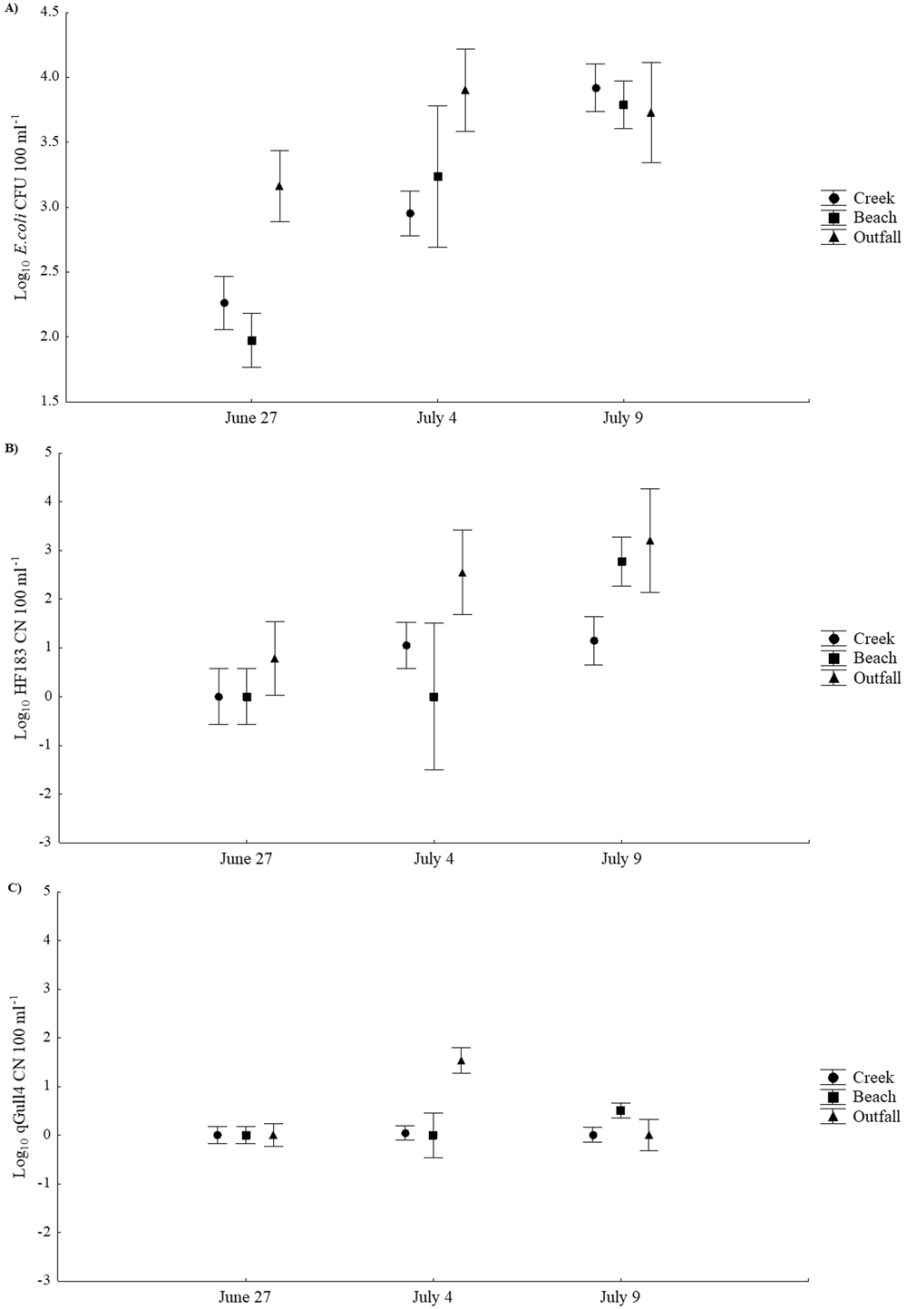
Mean concentrations (±SE) of (**A**) *E*. *coli*, (**B**) the MST human marker, and (**C**) the MSTgull marker in Etobicoke Creek, Marie Curtis Beach and stormwater outfalls across all sampling dates.

A list of the number of samples taken on each sampling date as well as the mean and standard deviation for all parameters measured can be found in Table 1. Based upon culture-based *E*. *coli* quantification, when samples from all three sampling dates were included, no significant difference was detected in *E*. *coli* concentrations among different site types (creek, beach or outfall). During the drier sampling dates on June 27^th^ and July 4^th^, *E*. *coli* concentrations (CFU 100 mL^−1^) were significantly higher in outfalls than at beach or creek sites(*F*_2,29_ = 6.98, *P* = 0.003; Tukey’s post-hoc test results were *P* = 0.04 and 0.002, respectively; Fig. 2A). Following the extreme rain event on July 9^th^, no significant differences were detected among site types with regard to *E*. *coli* concentrations (Fig. 2A). *E*. *coli* concentrations in creek and beach samples were significantly higher following the extreme rain event on July 9th (*F*_2,23_ = 38.27, *P* < 0.001 and *F*_2,14_ = 10.70, *P* = 0.002, respectively; Fig. 2A), although no significant difference was observed for *E*. *coli* concentrations in outfall samples among sampling dates.

**Table 1:** List of sampling dates, sites sampled on each date, as well as mean data for all parameters measure with standard deviations in parentheses.

Date	Sample Type	Number of Samples	Sites Sampled	log_10_ *E*. *coli* (CFU 100 mL^−1^)	Caffeine (ng L^−1^)	Carbamazepine (ng L^−1^)	Codeine (ng L^−1^)	Cotinine (ng L^−1^)	Acetaminophen (ng L^−1^)	qPCR			eDNA Sequences						% Agreement Between qPCR and eDNA for Human Detection
										log_10_ Human (CN 100 mL^−1^)	% Human Positive	log_10_ Gull (CN 100 mL^−1^)	Human	% Human Positive	Mammal	Frog	Bird	Fish	
6/27/2013	Creek	7	80002, 80006, BURN, DDAS, DERY, EGLI, WCRK	2.26 (0.54)	324.34 (355.05)	0.72 (0.10)	0	51.27 (7.95)	6.46 (6.27)	0	0	0	26.14 (28.42)	66.67	0	0	0	110.14 (92.73)	42.86
7/4/2013	Creek	10	80002, 80006, BURN, DDAS, DERY, EGLI, MALL, RB, SWAY, WCRK	2.95 (0.41)	435.26 (363.67)	1.05 (0.26)	0	34.34 (15.04)	53.57 (45.75)	1.05 (1.77)	30	0.03 (0.10)	36.90 (101.01)	60.00	1.0 (2.83)	0	1.80 (4.47)	64.50 (50.43)	50
7/9/2013	Creek	9	80002, 80006, BURN, EGLI, MALL, RB, SWAY, S1, WCRK	3.92 (0.11)	130.73 (86.53)	0.66 (0.86)	0	12.41 (8.75)	19.40 (24.44)	1.14 (1.79)	33.33	−0.06 (0.18)	2.56 (3.88)	55.56	4.78 (4.99)	1.33 (1.66)	1.22 (1.09)	8.44 (6.0)	55.56
6/27/2013	Beach	7	E.1, E.1.5, E.2, W.1, W.2, W.3	6.24 (0.99)	NT	NT	NT	NT	NT	0	0	0	9.67 (21.48)	28.57	0	0	0.33 (0.76)	15.50 (12.10)	71.42
7/4/2013	Beach	1	W.3	3.24 (0)	NT	NT	NT	NT	NT	0	0	0	3 (0)	100.00	0	0	0	21 (0)	0
7/9/2013	Beach	9	E.1, E.1.5, E.2, W.1, W.2, W.3	3.79 (0.58)	NT	NT	NT	NT	NT	2.76 (1.77)	77.75	0.45 (1.02)	2.56 (6.21)	44.44	0.67 (1.66)	0	1.56 (2.19)	13.11 (18.0)	44.44
6/27/2013	Outfall	4	COURTOF, DDASOF, GARNOF, SWAYOF	3.16 (0.50)	260.55 (408.91)	24.18 (44.27)	2.12 (4.23)	23.48 (10.90)	39.23 (40.14)	0.78 (0.155)	25	0	225.75 (440.24)	50.00	61.75 (122.17)	0	1.0 (2.0)	89.75 (128.17)	75
7/4/2013	Outfall	3	DDASOF, GARNOF, SWAYOF	3.90 (0.15)	7886.67 (10925.12)	1.21 (0.69)	0	173.37 (87.0)	1287.33 (1447.25)	2.55 (2.34)	66.67	1.51 (0.77)	30.33 (37.03)	100.00	70.33 (108.83)	0.67 (1.15)	180.67 (153.24)	4.67 (2.52)	66.67
7/9/2013	Outfall	2	DDASOF, EGLIOF	3.73 (0.36)	253.40 (217.22)	0.53 (0.65)	0	42.20 (44.55)	102.63 (134.88)	3.20 (0.46)	100	0	7.50 (7.78)	100.00	0.50 (0.71)	0	0	7.0 (9.90)	100

The percentage of samples positive for the human MST method and eDNA metabarcoding are also presented.

NT denotes that the parameter was not tested.

### Microbial Source Tracking

During the drier sampling dates a significant difference in concentrations of the MST markers was detected among site types (*F*_4,56_ = 3.26, *P* = 0.02; Fig. 2B,C), with Tukey’s post-hoc test revealing significantly higher concentrations of the human MST marker in outfalls than creek or beach sites (*P* = 0.008 and 0.017, respectively; Fig. 2B). Sampling date also had a significant effect on MST marker concentrations for beach sites (*F*_4,26_ = 3.34, *P* = 0.025), with concentrations of the human MST marker being significantly higher following the extreme rain event (Fig. 2B). There was also a significant difference in gull MST marker detection in stormwater outfalls (*F*_4,10_ = 7.96, *P* = 0.004), with significantly higher concentrations detected on July 4^th^ (Fig. 2C).

### Chemical Source Tracking

**Figure 3:**
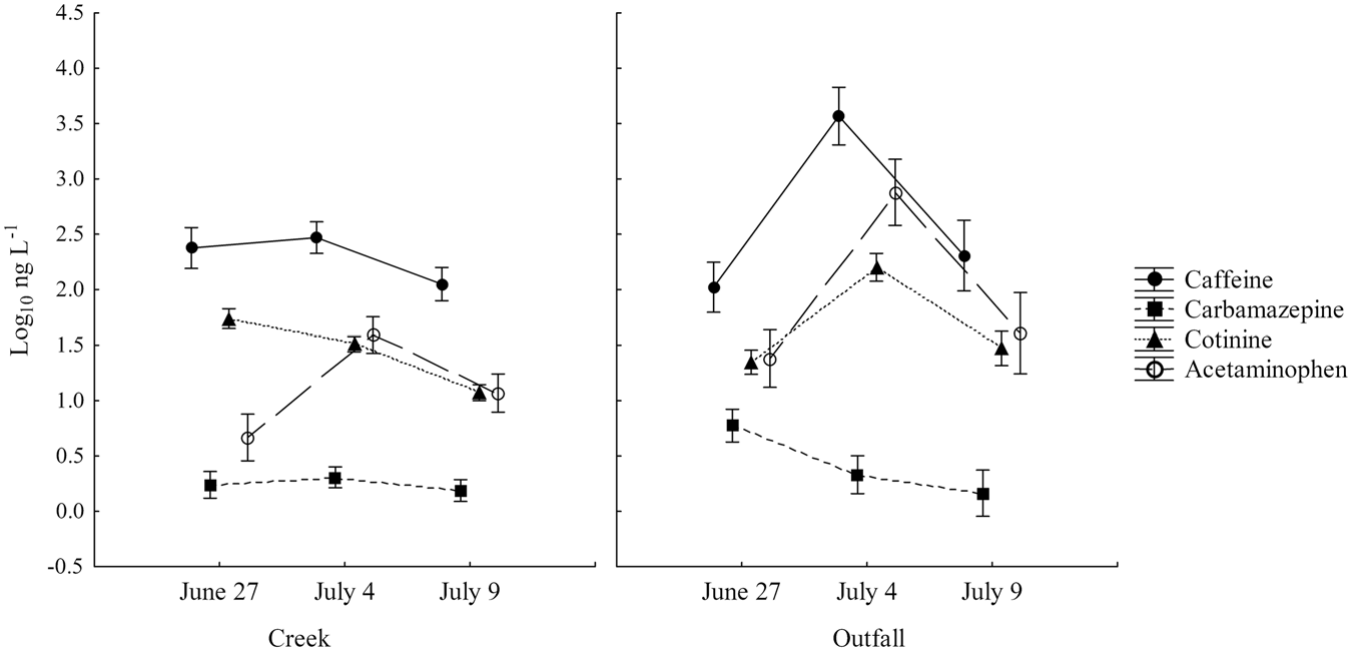
Mean concentrations (±SE) of caffeine, carbamazepine, cotinine and acetaminophen in Etobicoke Creek and stormwater outfalls across all sampling dates.

No significant difference in CST markers was detected among different site types (creek or outfall, as no CST analysis was done for beach sites). However, within creek samples, concentrations of cotinine were significantly lower on July 9^th^ than previous sampling dates (*F*_8,38_ = 13.20, *P* < 0.001; Fig. 3). Similarly, creek concentrations of caffeine and outfall concentrations of carbamazepine were also lowest on July 9th although this difference was not statistically significant.

### eDNA Metabarcoding

**Figure 4:**
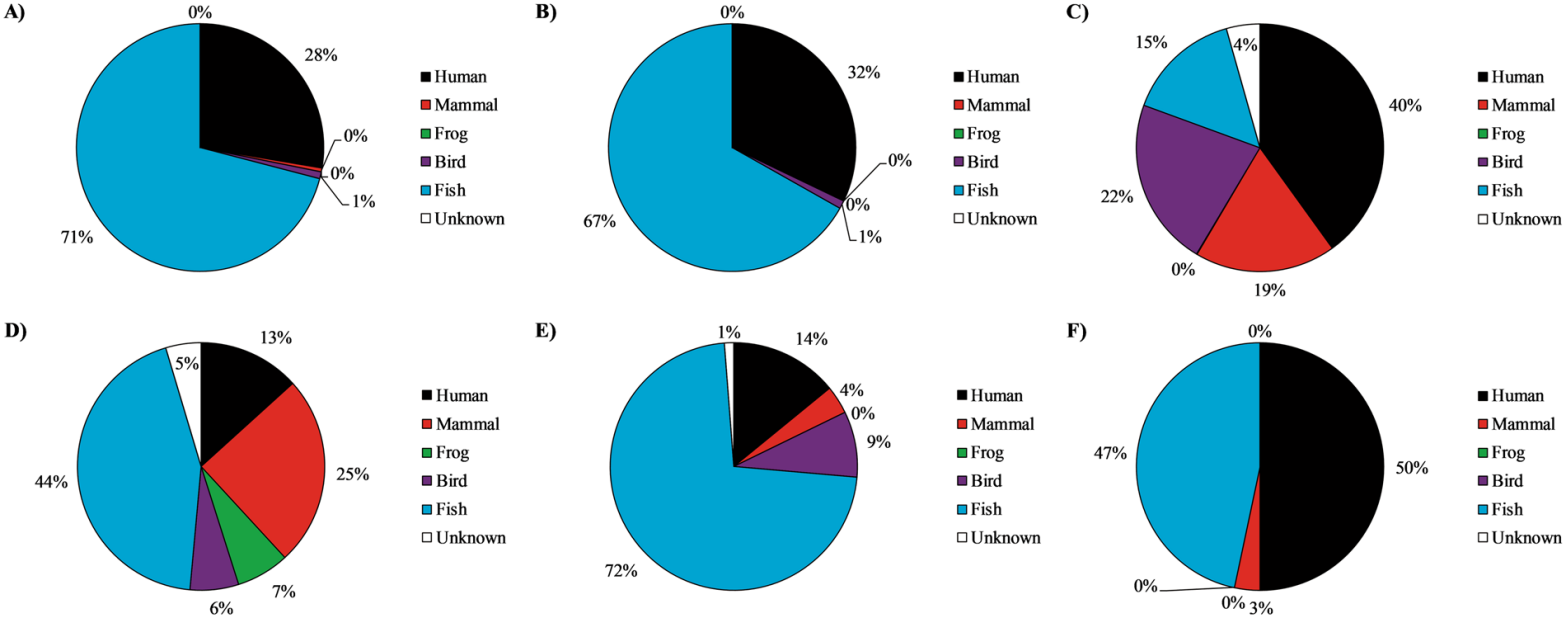
Percentage of sequences detected during dry sampling days in (**A**) Etobicoke Creek, (**B**) Marie Curtis Beach and (**C**) stormwater outfalls, and after the extreme rain event on July 9^th^ in (**D**) Etobicoke Creek, (**E**) Marie Curtis Beach and (**F**) stormwater outfalls. Percentages were calculated per sample type based upon the combined sequences of the drier sampling events compared to the percentages obtained following the extreme rain event.

The extreme rain event on July 9^th^ considerably altered the relative amount of eDNA sequences in all sample types compared to the drier June 27^th^ and July 4^th^ sampling dates (Fig. 4; percentages and the numbers presented below were calculated per sample type based upon the combined sequences of the drier sampling events compared to the percentages obtained following the extreme rain event). Within the creek, on drier days the majority of eDNA sequences (71%; 1416 of 1996 sequences) were identified as freshwater fish species, with the 2^nd^ highest abundance of sequences being identified as human (28%; 552 of 1996 sequences; Fig. 4A; Table 2). In addition, 9 gull (*Larus delawarensis*) sequences were detected on July 4^th^. However, following the extreme rain event of July 9^th^, the relative abundance of fish and human sequences were reduced to 45% (78 of 173 sequences) and 13% (23 of 173 sequences), respectively, while the diversity and number of non-human mammal eDNA sequences increased to comprise 25% (43 of 173 sequences) of the total sequence reads detected (Fig. 4D; Table 2). This included eDNA from urban wildlife, such as Eastern gray squirrel, meadow vole, and Red-winged blackbird (*Sciurus carolinensis*, *Microtus pennsylvanicus*, *Agelaius phoeniceus*, respectively) and cat (*Felis catus*) sources that were not detected on the drier days. Of note, *Gallus gallus* (chicken) and *Bos* (cow) species sequences were also detected in the creek only on July 9^th^ (Table 2).

**Table 2:** Number of sequences as each animal source in each sample type (creek, beach or outfall) across all sampling dates.

Category	Animal Source	Creek			Beach			Outfall		
		27-Jun	4-Jul	9-Jul	27-Jun	4-Jul	9-Jul	27-Jun	4-Jul	9-Jul
Human	*Homo sapiens*	183	369	23	58	3	23	903	91	15
Mammal	Bos*			37				243	191	
	*Sciurus carolinensis*			1					6	
	*Tamiasciurus hudsonicus*								1	
	*Marmota monax*						1			
	*Ondatra ziberthicus*		9							
	Tamis*								1	
	*Tamis striatus*								3	
	Peromyscus*							2		
	*Microtus pennsylvanicus*			1						
	*Procyon lotor*		1	3			5	2	9	1
	*Felis catus*			1						
	Total Mammal	0	10	43	0	0	6	247	211	1
Bird	*Passer domesticus*								10	
	*Turdus migratorious*								1	
	*Agelaius phoeniceus*			2	14				1	
	*Pooecetes gramineus*			2						
	Larus*								4	
	*Larus delawarensis*		10						307	
	*Gallus gallus*			7			8		185	
	*Meleagris gallopavo*								34	
	Anas*		8							
	*Branta canadensis*				2		6	4		
Frog	Total Bird	0	18	11	16	0	14	4	542	0
	Anaxyrus*			2					2	
	*Anaxyrus americanus*			4						
	*Rana clamitans*			6						
	Total Frog	0	0	12	0	0	0	0	2	0
Fish	*Luxilus cornutus*	103	234	23			1			9
	*Pimephales promelas*	1	11	5			1	145		
	*Pimephales notatus*	122	33	5						
	*Rhinichthys cateractae*	101	100	27	2	2		53	9	
	*Rhinichthys obtusus*	344	201	6				16		5
	*Catostomus commersonii*	8	9	3			1			
	*Notropis atherinoides*				12	19	83	10		
	*Campostoma anomalum*	73	9	2						
	*Semotilus atromaculatus*	19	23	1						
	Dorosoma*						4			
	*Dorosoma cepedianum*				1		8	1		
	Alosa*				75		13	7		
	*Alosa pseudoharengus*				15			6		
	Oreochromis*								2	
	Serranidae*			128					2	
	*Salmo salar*								1	
	*Oncorhynchus nerka*							4		
	*Oncorhynchus tshawytsch*				1					
	Oncorhynchus*							14		
	*Neogobius melanostomus*		22				6			
	Etheostoma*		1	1						
	*Culaea inconstans*			3				30		
	*Ambloplites rupestris*		1							
	Lepomis*			2				60		
	*Lepomis cyanellus*				1			13		
	*Micropterus dolomieu*		1							
	Total Fish	771	645	206	107	21	117	359	14	14
Ambiguous	Ambiguous			6			3	64	45	
	Total Sequences	954	1042	173	167	24	163	1577	905	30

*Sequence only identified to the genus level.

Among beach samples, most eDNA sequences were identified as fish species (63%; 121 of 191 sequences) or human sequences (35%; 61 of 191 sequences) in the drier sampling dates (Fig. 4B; Table 2). Following the extreme rain event, the beach samples saw a reduction in the relative abundance of human sequences (to 14%; 23 of 163 sequences), with an increase in diversity and number of non-human mammal and bird eDNA sequences (4%; 6 of 163 sequences and 9%; 14 of 163 sequences, respectively), although the relative amount of fish eDNA sequences remained relatively unchanged (72%; 117 of 163 sequences compared to 63%; 121 of 191 sequences: Fig. 4E; Table 2). Canada geese (*Branta canadensis*) eDNA was detected more often from water samples at the beach than from the creek or stormwater. Similar to creek samples, *Gallus gallus* (chicken) sequences were only detected in the beach samples on July 9^th^ (Table 2).

During the drier sampling dates, eDNA sequences in outfalls were predominately human (40%; 994 of 2482 sequences) followed by bird (22%; 546 of 2482 sequences) and mammal sequences (18%; 458 of 2482 sequences; Fig. 4C; Table 2). However, during the extreme rain event, the diversity of eDNA sequences in stormwater outfalls was reduced and they were almost exclusively human (50%; 15 of 30 sequences) and fish (47%; 14 of 30 sequences). eDNA from raccoon (*Procyon lotor*) was most common in stormwater and was detected on each sampling date. Of particular note, among the fish species detected on July 4^th^ in the outfall samples were sequences identified as *Oreochromis* spp, *Serranidae* spp., and *Salmo salar* (non-native food fish; Table 2).

**Figure 5:**
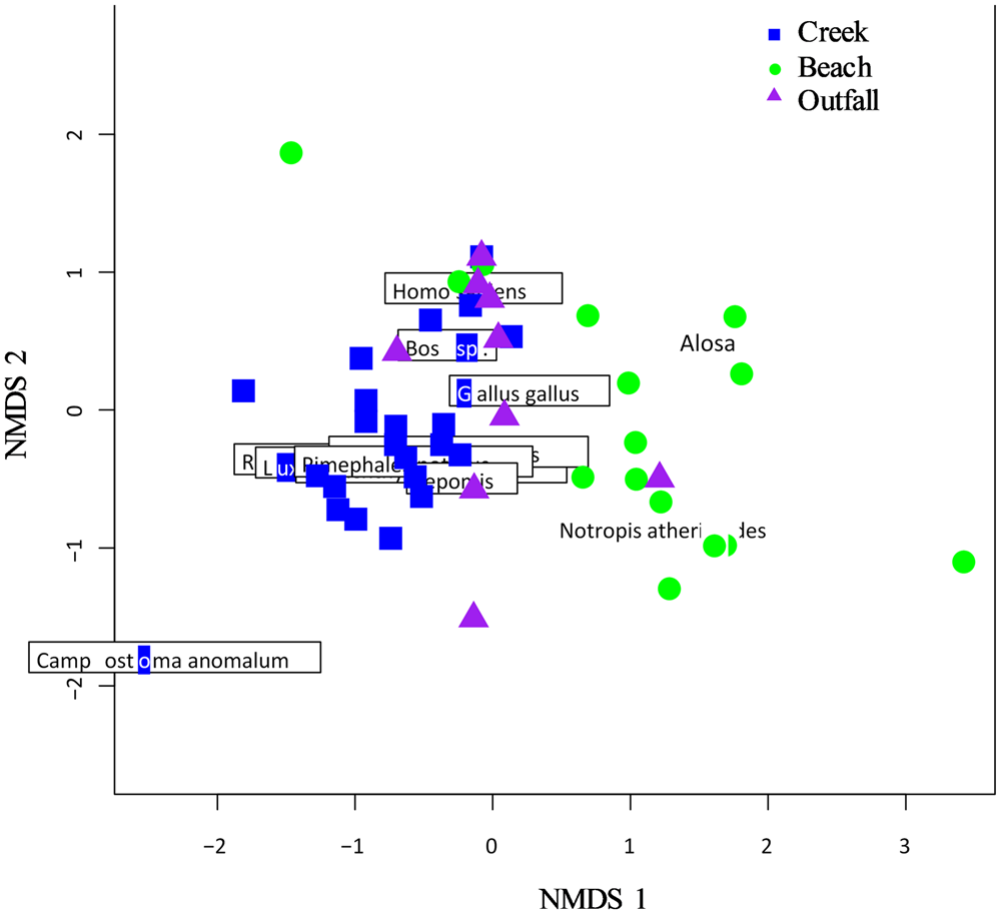
Non-metric multi-dimensional scaling analysis shows that communities within sample subtypes are similar. Sites are indicated by colored symbols (blue squares = Creek samples, green circles = Beach samples, purple triangles = Outfalls). Only unambiguously identified taxa that comprised more than 1% of the reads are shown. Only the first two axes are shown.

The first two dimensions of the NMDS (non-metric multidimensional scaling) plot are shown in Fig. 5 (stress = 0.09). The goodness of fit between the Bray Curtis distances and the ordination distances are positively correlated (non-metric R^2^ = 0.992). The goodness of fit for the fitted factor subtype (Creek, Beach and Outfalls) were positively correlated with the ordination distances (R^2^ = 0.38, *P* = 0.001). In the NMDS plot, only identified OTUs comprised of greater than 1% read abundance are shown. The community composition for each subtype tends to cluster together showing that they are similar.

### Associations among FST

Significant correlations were observed among all site types. With regard to creek samples, concentrations of the human MST marker were significantly correlated with concentrations of acetaminophen (*r*_*s*_ = 0.46) and the MST gull marker (*r*_*s*_ = 0.58). Among beach samples, concentrations of *E*. *coli* were significantly correlated with concentrations of the human MST marker (*r*_*s*_ = 0.76); concentrations of the human MST marker were significantly correlated with concentrations of the gull MST marker (*r*_*s*_ = 0.65). Within stormwater outfall samples, *E*. *coli* concentrations were significantly correlated with concentrations of cotinine (*r*_*s*_ = 0.87) and acetaminophen (*r*_*s*_ = 0.83).

Among all samples, concentrations of the human MST marker were significantly correlated with the number of human eDNA sequence reads detected (*r*_*s*_ = 0.73), although human eDNA sequences were detected in the absence of the human MST marker at many sites. Among creek samples, 42% had detection of human eDNA sequences without detection of the human MST marker; by contrast only 8% had detection of the human MST marker without detection of a human eDNA signature. Among outfalls, human eDNA sequences were detected in 22% of samples without a human MST marker detect, though the human MST marker was never detected without there also being detection of a human eDNA sequence. In beach samples, a human eDNA sequence was detected in 24% of samples with no corresponding detection of a human MST marker; conversely, 24% of samples also had detection of the human MST marker with no corresponding human eDNA sequence detected. When samples from all site types were combined, a human eDNA sequence was detected 70.6% of the time that the human MST marker was detected; by contrast, the human MST marker was detected only 42.9% of the time a human eDNA sequence was detected. Coherence between qPCR and eDNA detection for each site type and date are listed in Table 1. While a high number of human eDNA sequence reads tended to also result in human MST marker detection among stormwater outfalls, this relationship was not true of creek or beach sites. With regard to the gull MST marker, 10% of total samples had gull MST marker detection with no corresponding detection of gull eDNA sequences, whereas gull eDNA sequences were never detected without also detecting the gull MST marker.

## Discussion

This study found a significantly altered fecal pollution profile in an urban creek, associated stormwater outfalls, and an urban beach associated with an extreme rain event. Stormwater outfalls within the watershed were prominent sources of fecal contamination, with the highest levels of both human and gull MST markers, particularly on the July 4^th^ sampling date. Additionally, eDNA profiling found the highest percentage of human sequences in outfalls relative to creek or beach sites. The extreme rain event on July 9^th^, where ~126 mm of rain fell 24 hours prior to sampling, had profound effects on the watershed with regard to *E*. *coli* and FST marker concentrations. *E*. *coli* concentrations were significantly higher in creek and beach sites, nearing a mean of 10,000 *E*. *coli* CFU 100 mL^−1^ in all site types. Concentrations of the human MST marker were also significantly higher in stormwater outfalls and at the beach sites. The percentage of human eDNA sequences in stormwater outfalls also increased to encompass 50% of sequences detected. These results are similar to other studies which have found correlations between rainfall and increased concentrations of *E*. *coli* and MST markers^31–34^. By contrast, concentrations of most CST markers tended to decline in both creek and outfall samples, possibly as a consequence of dilution (Fig. 3).

While the effects of rainfall on *E*. *coli* and MST marker detection and concentrations have been well investigated, this is among the first studies to examine the effects of an extreme rain event on eDNA profiles, which changed considerably relative to drier sampling dates. Following the extreme rain event, on July 9^th^ the creek and beach samples had more diverse eDNA profiles than during their drier sampling dates, with relatively more sequences from mammal and bird species which were absent during dry sampling dates. eDNA from urban animals like grey squirrel (*Sciurus carolinensus*), groundhog (*Marmota monax*), vole (*Microtus pennsylvanicus*), cat (*Felis catus*), sparrow (*Pooecetes gramineus*), toad (*Anaxyrus americanus*), and frog (*Rana clamitans*) were only detected in water samples following the extreme rain event. Similarly, eDNA from cow (*Bos* sp.) and chicken (*Gallus gallus*) were only detected in creek and beach surface waters following the extreme rain event.

The Etobicoke Creek watershed currently consists of three major land uses: 68% urban, and 5% urbanizing, and 27% rural^35^. While there are small numbers of livestock (beef and dairy cattle) and one broiler chicken farm in the uppermost rural reaches of the Creek^36^, other nonpoint sources of contamination from urbanization are considered the largest contributor to water quality impairments^35^. However, the extreme rain event could potentially have brought cattle and chicken fecal contamination down to Lake Ontario and the associated beach sites during large storm events. Alternatively, several meat processing plants are located in the more urbanized lower portion of Etobicoke Creek, which may be more likely explanations of the bovine and chicken eDNA signatures.

Outfall samples showed a different rain event response from the creek and beach, with less diverse eDNA profiles and fewer eDNA reads that were almost exclusively from human and fish sources following the extreme rain event; only a single raccoon eDNA sequence was otherwise detected. These changes suggest that the large amount of rainfall diluted some sources in the outfalls, as likely happened with concentrations of the CST markers. Additionally, the discrepancy in diversity and eDNA reads between the July 4^th^ and July 9^th^ events could be due to more rapid flushing of the storm-sewer systems (on July 9^th^). Extreme rainfall associated with more impervious surfaces likely resulted in unusually high stormwater flows that could have diluted eDNA signatures from smaller fecal sources. The opposite response in creek samples, of higher eDNA diversity associated with the extreme rain event, could have resulted from extensive flooding and novel overland run off pathways to the creek. eDNA sequences in terrestrial soil and streambed sediment reservoirs may also have been released into the creek associated with erosion and scouring from the extreme rain event.

Interestingly, during the drier sampling dates, the outfalls had more diverse eDNA profiles, with eDNA from food animals like cow (*Bos* sp.), chicken (*Gallus gallus*), Atlantic salmon (*Salmo salar*), Sea Basses (Serranidae sp.), and Tilapia (*Oreochromis* sp.) only detected on these dates. The sequences of *Oreochromis* spp., *Serranidae* spp., and *Salmo salar* – species of food fish not expected to be resident within the watershed – were detected within outfall samples and are likely representative of fish that were eaten or intended for consumption. Similarly, the sequences of food animals like cow (*Bos* spp.) and chicken (*Gallus gallus*) were not expected fecal sources in our urban area. It is unclear whether these food animal sequences originated from a fecal source (including digested food items passing into human sewage) or an abattoir or food processing source within the stormwatershed.

While eDNA analysis can be useful in detecting both major and minor sources of fecal contamination, caution should be used when interpreting these results, as eDNA sequences may not be of fecal origin. In addition to fecal origin, eDNA can also be shed through urine, saliva, blood or skin and may therefore not be indicative of fecal contamination^37,38^. Consequently, human eDNA sequences detected on beach sites may be the result of epithelial shedding from swimmers rather than fecal contamination. Additionally, a carryover effect has been suggested as a consequence of diet, as several studies have reported obtaining a bovine or chicken eDNA signature from feces of human participants who had recently eaten beef or chicken, respectively^37,39^. However, it should be noted that other studies have not observed this carryover effect^38,40^.

Whereas MST markers are useful for identifying specific sources of fecal contamination and CST markers may provide additional insight into sewage contamination, eDNA data could provide a more comprehensive profile of the possible fecal pollution sources present within a watershed. However, it should be noted that not all species identified via eDNA sequencing are necessarily problematic sources of fecal contamination. For example, within the Etobicoke Creek watershed, the predominant fish species detected within the creek were *Luxilus* and *Rhinicthys* species, and in beach samples, *Notropis* and *Alosa* species – all freshwater fish which would be expected within this watershed study area, and within these respective creek and lake habitats. Of value, however, are detection of eDNA sequences of urban wildlife like muskrat and raccoon found within creek samples, which likely are contributors to *E*. *coli* concentrations^41,42^. Additionally, within outfall sites, eDNA sequence data and concentrations of the human MST marker were significantly correlated and, among all samples, human eDNA sequences were detected 70% of the time the MST human marker was detected, suggesting that eDNA can be a useful tool in detection of sources of sewage contamination. However, human eDNA was detected in the absence of the human MST marker at many sites, particularly creek sites, suggesting caution, as human eDNA sequences could result from non-fecal human contamination (i.e. from skin). A similar comparison between the eDNA data and the gull MST marker was less reliable, as this gull marker has been found to cross-react with other bird species, particularly waterfowl, so increased qPCR detection may not necessarily correspond only to gull-specific fecal contamination^43^.

The results of this study provide a proof of concept to suggest that metabarcoding of bird, mammal, and human eDNA sequences from water samples can be a useful complement to microbial and chemical source tracking tools in understanding sources of fecal contamination in aquatic ecosystems. However, this study had several limitations, largely owing to a limited sample size. Additionally, no negative controls (i.e. field blanks or DNA extraction blanks) were sequenced for eDNA metabarcoding to be able to rule out contamination from human epithelial cells during sampling processing. Further, only one major rain event was sampled and not all sites were sampled during all three sampling events, limiting the extent to which our conclusions can be used to predict future outcomes.

The results of this study also demonstrate that while all FST methods attempt to remedy the imperfections of solely using FIB to assess water quality, each method has specific drawbacks. MST qPCR methods are largely limited in that each individual assay can only detect a singular known target. CST methods may only be applicable as a predictor of human sewage contamination. The use of eDNA metabarcoding, while advantageous in that it can identify multiple targets directly without requiring a priori knowledge of fecal sources, may provide a profile which is not reflective of exclusively fecal contamination. Additionally, while eDNA metabarcoding can provide a considerable amount of information, this method is considerably more costly and time consuming than using qPCR assays, particularly when a specific source of fecal contamination is likely in a given watershed.

The results of this study indicate the profound effect that extreme rain events can have on the concentrations of fecal contaminants as well as the eDNA profiles of an impacted watershed. With the onset of climate change, the frequency and magnitude of extreme rainfall events are expected to increase^44–47^, with one study projecting that this can lead to an increase in waterborne disease risk for recreational and drinking waters^48^. As the rainfall event in this study has shown, extreme rainfall events also contribute a diverse amount of DNA and potential contaminants into nearby water bodies, which may also have implications for ecological health and the risk of disease for other aquatic life. Further research is needed to understand how these rain events influence the delivery and distribution of fecal contamination in aquatic ecosystems.

## Methods

This study was conducted in the Etobicoke Creek watershed and at Marie Curtis Beach at the mouth of the creek (Toronto, ON). Water samples were collected in the morning on June 27^th^, July 4^th^, and July 9^th^, 2013. The only rainfall in the preceding 72 hours for the June 27^th^ sampling date was 4.2 mm of rain that fell 48 hours prior to water sample collection. The only rainfall in the preceding 72 hours for the July 4^th^ sampling date was 1.2 mm of rain that fell 24 hours prior to water sample collection. As these rainfall amounts were less than the 5 mm often used by the city of Toronto to consider stormwater responses, we refer to these dates as drier sampling dates. The July 9^th^ sampling date followed an extreme rain event where ~126 mm of rain fell within the preceding 24 hours, mostly in a two hour period between 4:30 and 6:30 pm. Water samples were collected in autoclaved 500 ml polypropylene bottles from 11 sites along Etobicoke Creek (*n* = 26), five associated stormwater outfalls (*n* = 9) and six transects along Marie Curtis beach (*n* = 17) consisting of ankle- and chest-depth samples within Lake Ontario (a map of all sampling locations can be found in Fig. 1). Simultaneously, water samples were taken for chemical analysis in a 100 ml amber glass bottle from all creek and outfall samples. All water samples were placed on ice and transported back to the lab for processing within six hours of collection. Data on rainfall the day of sampling and 24-, 48-, and 72-hours prior to sampling was obtained for the Pearson International Airport station, located within the Etobicoke Creek watershed, from the Environment and Climate Change Canada website (http://climate.weather.gc.ca/historical_data/search_historic_data_e.html). For enumeration of *E*. *coli*, water samples were filtered (0.45 µm pore size, 47 mm diameter, nitrocellulose membranes) over a range of dilutions according to standard membrane filtration methods^49^ and incubated at 44.5 °C for 22 hours on differential coliform media (Oxoid Inc., Hampshire, UK), supplemented with cefsulodin. Additionally, 300 ml was also filtered (0.45 µm pore size, 47 mm diameter, nitrocellulose membranes) for each sample and the entire filter was used for DNA extraction, ground up in a PowerBead™ tube using flame-sterilized forceps. DNA was extracted using Powersoil™ DNA Isolation Kits (MO BIO Laboratories, Inc., Carlsbad, CA, USA) according to manufacturer’s instructions.

### Quantitative Polymerase Chain Reaction

Quantitative polymerase chain reaction (qPCR) assays included human (HF183) and gull (qGull4) assays, using previously published primer sets^50,51^. Each qPCR reaction consisted of 2 µl of an internal amplification control (IAC), 2.5 µl 2 mg ml^−1^ BSA, 3 µl nuclease-free water, 12.5 µl TaqMan® Universal Master Mix 2.0 (Applied Biosystems, Carlsbad, CA, USA), 3 µl of a primer/probe mixture (920 nM final concentrations for all primers and 76 nM final concentration for all probes), and 2 µl of extracted DNA. Reactions were carried out in 96-well plates using a Bio-Rad CFX96 cycler (Hercules, CA, USA). All reactions were carried out in duplicate, including non-template controls, negative controls consisting of 2 µl salmon testes DNA, and positive controls consisting of 2 µl of DNA extracted from a known fecal source. Thermocycler conditions with the same as in previously published assays^50,51^.

Standard curves for all qPCR assays were constructed using synthesized plasmid DNA (pIDTSMART with ampicillin resistance; Integrated DNA Technologies, Coralville, IA, USA). DNA used for the standard curve was serially diluted using AE buffer (Qiagen, Valencia, CA, USA) to concentrations ranging from 10^2^ to 10^5^ gene copies reaction^−1^. DNA used for the IAC was similarly constructed using synthesized plasmid DNA (pIDTSMART with ampicillin resistance; Integrated DNA Technologies, Coralville, IA, USA) with complementary primer sites for each assay and included in every reaction to verify that there was no inhibition. All qPCR runs had an efficiency between 90 and 110% with and R^2^ of >0.95 and results were normalized to reaction efficiency. Results were reported as copy number (CN) 100 mL^−1^.

### Chemical Source Tracking Analysis

CST analysis for caffeine, carbamazepine, codeine, cotinine and acetaminophen was performed on all creek and stormwater outfall samples. No CST analyses was performed on beach samples due to cost limitations. Analyses were performed by Environment and Climate Change Canada’s National Laboratory for Environmental Testing (Burlington, ON,). In brief, a Waters Xevo TQ-S system was used to separate the compounds which were then analyzed by a Waters Xevo TQ-S UHPLC-MS/MS with an Electrospray Ionization (ESI) source utilizing polarity switching. The full methods have been previously described^49^. For quantitative analysis, where 50% of samples for a particular site were above the limit of detection (LOD), then ½ LOD was assigned in the event of a non-detect; if less than 50% the samples for a particular site were above the LOD, then a value of 0 was used. This method was only used for CST data.

### Amplification and eDNA Library Preparation

A total of 52 DNA samples were sequenced in a single MiSeq experiment. Two fragments within the standard COI DNA barcode region were amplified with two indexed primer sets in a two-step PCR amplification regime^52^. The first sequenced fragment is called F230 which is 230 bp in length and is found at the 5′ end of the standard barcoding region. The amplification primers of this region are, the standard Folmer *et al*. forward primer (F GGTCAACAAATCATAAAGATATTGG)^53^ and the universal reverse primer (230_R CTTATRTTRTTTATICGIGGRAAIGC)^54^. The second fragment is BR5 fragment of *COI* which is approximately 314 bp in length, is found toward the 3′ end of the standard barcoding region. The BR5 fragment is amplified using the following primers, B CCIGAYATRGCITTYCCICG^52^, and R5 GTRATIGCICCIGCIARIAC^55^.

The first PCR used *COI* specific primers and the second PCR involved Illumina-tailed indexed primers. The *COI* primers were selected because these primers are highly degenerate and have been used efficiently for their wide coverage between many taxonomic groups^55,56^. The PCR reactions were assembled in 25 μL volumes. Each reaction contained 17.5 μL molecular biology grade water, 2 μL DNA template, 2.5 μL 10X reaction buffer (200 mM Tris HCl, 500 mM KCl, pH 8.4), 1 μL MgCl_2_ (50 mM), 0.5 μL dNTPs mix (10 mM), 0.5 μL forward primer (10 mM), 0.5 μL reverse primer (10 mM), and 0.5 μL Invitrogen’s Platinum Taq polymerase (5 U μL^−1^). The PCR cycling conditions were initiated with heated lid at 95 °C for 5 min, followed by a total of 25 cycles of 94 °C for 40 s, 46 °C for 1 min, and 72 °C for 30 s, and a final extension at 72 °C for 5 min, and hold at 4 °C. Amplicons from each sample were purified using Qiagen’s MiniElute PCR purification columns and eluted in 30 μL molecular biology grade water. Subsequently, the purified amplicons from the first PCR were used as templates in the second PCR with the same amplification condition using Illumina-tailed indexed primers in a 12-cycle amplification regime. All PCR product quantification steps were performed by fluorometer and all PCRs were done using Eppendorf Mastercycler ep gradient S thermocyclers and negative control reactions (no DNA template) were included in all runs. PCR products were visualized on a 1.5% agarose gel to check the amplification success.

All generated amplicons were purified in Qiagen’s MiniElute PCR purification columns and eluted in 30 μL molecular biology grade water. The pooled equimolar amplicons were dual indexed using Illumina-Nextera 96 indexed kits for 10 cycles and pooled into a single tube to be sequenced on a MiSeq flowcell using a V3 MiSeq sequencing kit (2 × 300; MS-102-1003).

### Bioinformatic Processing and Data Analysis

Each sample was sequenced using approximately 50,000 sequence reads per sample. The generated sequences were de-multiplexed using the default parameters of Illumina MiSeq control software. Then for each sequencing pool, the forward and reverse raw reads were merged with SEQPREP software (https://github.com/jstjohn/SeqPrep) requiring a minimum overlap of 25 bp and no mismatches. All Illumina paired-end reads were then de-multiplexed again based on the indexes and the amplification primers and filtered for quality using PRINSEQsoftware^57^ with a minimum Phred score of 20, window of 10, step of 5, and a minimum length of 100 bp. USEARCH v6.0.307^58^. The UCLUST algorithm for OTU clustering was used to de-replicate and cluster the passing filters sequences using a 99% sequence similarity cutoff to denoise any possible sequencing errors prior to further processing. Chimera filtering was performed using USEARCH with the ‘de novo UCHIME’ algorithm^59^. At each step, cluster sizes were retained. Singletons were retained as our analysis is based on the taxonomical identification, so keeping the singleton and assigning them to lowest common ancestor with our strict parameters maximize the use of all generated data and avoid any data loss due to minor PCR or sequencing artifacts, and only putatively non-chimeric reads were retained for further processing. Additionally, paired-end sequencing was done to produce minimal sequencing errors. The retained clusters of both F230 and BR5 of each sample were pooled and identified using the MEGABLAST algorithm^60^ against a reference library. This reference library contained all verified *COI* sequences downloaded from the GenBank database January 15, 2015 with a minimum length of 100 bp. All MEGABLAST searches were conducted with a minimum alignment length percentage of 85% and a minimum similarity of 90%. Taxonomic identifications were recovered based on unambiguous top matches using all curated records from the NCBI/BOLD for the target gene regions. Species, genus, family, and order matrices for taxa with a minimum of ten sequences per cluster were generated for each sample based on these matches. We have used the NCBI taxonomy database to the LCA based on the sequence similarity% and query coverage% to the NCBI/BOLD database. All sequence data have been deposited into GenBank (accession numbers will be provided later).

### Statistical Analysis

All response variables (*E*. *coli*, CST, and MST concentrations) were log-transformed prior to analysis. Analysis of Variance (ANOVA) was used to determine the main effect of sample type (creek, beach or outfall) or sampling date, where response variables were *E*. *coli* concentrations. Multiple Analysis of Variance (MANOVA) was similarly used to assess main effects of site type or sampling date, with response variables being concentrations of the human and gull DNA markers or concentrations of caffeine, carbamazepine, cotinine and acetaminophen (codeine was never detected in any sample). Tukey’s post-hoc test was performed when significant differences were detected. Each model had only one explanatory variable, either sample type or date. Spearman correlations were used to assess relationships among *E*. *coli*, MST marker, and CST marker concentrations. The above analyses were performed in Statistica v.12, and results were considered significant at the α-level of 0.05.

In R, variable library sizes were normalized by converting to proportions: each taxon read count was divided by the total number of reads per sample (row totals). We only included taxa that were unambiguously identified. A NMDS plot was created using the R package VEGAN^61^ using the ‘metaMDS’ function with the Bray Curtis dissimilarity metric, k = 3 dimensions, and trymax = 100. Goodness of fit between the observed (Bray Curtis dissimilarities) and NMDS ordination distances were assessed using the ‘goodness’ function. Goodness of fit between the observed and fitted environmental variables (Date, Subtype) were assessed using the ‘envfit’ function and significance was assessed using 999 permutations.
